# Use of Connected Technologies to Assess Barriers and Stressors for Age and Disability-Friendly Communities

**DOI:** 10.3389/fpubh.2021.578832

**Published:** 2021-03-11

**Authors:** Preeti Zanwar, Jinwoo Kim, Jaeyoon Kim, Michael Manser, Youngjib Ham, Theodora Chaspari, Changbum Ryan Ahn

**Affiliations:** ^1^Center for Population Health and Aging, School of Public Health, Texas A&M University, College Station, TX, United States; ^2^Center for Health Systems and Design, Colleges of Architecture and Medicine, Texas A&M University, College Station, TX, United States; ^3^Network on Life Course and Health Dynamics and Disparities, University of Southern California, Los Angeles, CA, United States; ^4^Department of Multidisciplinary Engineering, College of Engineering, Texas A&M University, College Station, TX, United States; ^5^Department of Construction Science, College of Architecture, Texas A&M University, College Station, TX, United States; ^6^Texas A&M Transportation Institute, Texas A&M University System, College Station, TX, United States; ^7^Department of Computer Science and Engineering, College of Engineering, Texas A&M University, College Station, TX, United States

**Keywords:** stressors, connected technologies, wearable sensors, computer vision, transport technologies, alternative transport modes, age-friendly communities, disability-friendly communities

## Abstract

**Background:** The benefits of engaging in outdoor physical activity are numerous for older adults. However, previous work on outdoor monitoring of physical activities did not sufficiently identify how older adults characterize and respond to diverse elements of urban built environments, including structural characteristics, safety attributes, and aesthetics.

**Objective:** To synthesize emerging multidisciplinary trends on the use of connected technologies to assess environmental barriers and stressors among older adults and for persons with disability.

**Methods:** A multidisciplinary overview and literature synthesis.

**Results:** First, we review measurement and monitoring of outdoor physical activity in community environments and during transport using wearable sensing technologies, their contextualization and using smartphone-based applications. We describe physiological responses (e.g., gait patterns, electrodermal activity, brain activity, and heart rate), stressors and physical barriers during outdoor physical activity. Second, we review the use of visual data (e.g., Google street images, Street score) and machine learning algorithms to assess physical (e.g., walkability) and emotional stressors (e.g., stress) in community environments and their impact on human perception. Third, we synthesize the challenges and limitations of using real-time smartphone-based data on driving behavior, incompatibility with software data platforms, and the potential for such data to be confounded by environmental signals in older adults. Lastly, we summarize alternative modes of transport for older adults and for persons with disability.

**Conclusion:** Environmental design for connected technologies, interventions to promote independence and mobility, and to reduce barriers and stressors, likely requires smart connected age and disability-friendly communities and cities.

## Introduction

Nearly one-fourth percent of the United States (U.S.) population is an older adult; one-fifth have a disability (1). The population of the older adults in the U.S. is projected to increase to 94.7 million by 2060 (1). About 90% of the older population would prefer to age in their homes and communities instead of institutional settings (1). Outdoor physical activity has multitudinous advantages for older adults (2). Physical activity can help reduce the risk of mental health problems and physical disease, such as depression, obesity, cancer, and cardiovascular diseases (2). Maintenance of safe mobility is essential for successful aging in communities and a major challenge faced by older adults. Those with limitations cease driving and depend on their caregivers, informal supports and services, or other alternative modes of transport to stay connected and mobile. For older adults to stay mobile, it is essential their physical and service environment is stressor and barrier free. Therefore, monitoring of outdoor physical activities is an area of high priority and a need (3).

While benefits of engaging with environment are numerous, such built environments can impose numerous barriers and stressors for older adults and for persons with disabilities to age-in-place. Prior studies/reviews have been conducted various demographic groups, such as older adults (4, 5), premenopausal women (6), children (7), and obese adults (8) to examine the relationship between urban elements (e.g., transportation systems, neighborhood disorders, land use) and their behaviors (9). However, prior studies could not sufficiently identify how older adults respond to diverse elements of urban built environments, including structural characteristics, safety attributes, and aesthetics. No prior review has explored the variety of stressors and barriers that can hinder healthy aging in one's preferred environments. Our review was aimed to fill this gap. The rational was 2-fold: (i) to assess the use of physiological responses to identify how older adults react to their ascribed environments during outdoor physical activity using physiologic responses, and to identify the (ii) needs of communities to adapt to the needs of older adult's so they can age-in-place.

We know connected technologies have numerous benefits and potential to allow older adults and those with disabilities to facilitate safe mobility, reduce falls and allow for partaking in outdoor physical activity. Traditional technologies have become outdated and emerging technologies are rapidly evolving. The purpose of this mini-review was to synthesize and describe the use of three emerging connected technologies to mitigate barriers and stressors to environmental stimuli for older adults and those with disabilities from a multidisciplinary perspective (e.g., from population health and aging, to health systems and design, to life-course health dynamics and disparities, to computer science and multidisciplinary engineering, to construction, architecture and transportation science), with the motivation for older adults and those with disabilities of all ages to successfully age in their neighborhoods and in their community and in their city environments. We defined connected or smart technology as embedded technology with sensors, processors, camera, and location services that would allow connection and communication with its environment *via* internet of things and provide data that could be accessed and analyzed *via* a platform. Additionally, the review provides challenges of collection, processing and analysis of mobile, real-time connected data from smartphones in various populations, including older adults, and how such data can be aggregated and visualized.

## Methods

We conducted a literature review with no date restriction on connected technologies for older adults and for those with disability. We focused on three key connected technologies to assess stressors and barriers in community environments: (1) wearable sensing technologies; (2) computer vision techniques; and (3) transport needs, technologies and options for alternative transport modes. We used the following keywords “wearable sensing,” “physiological signals,” “physical activity or disorders,” “urban built environment,” “environmental barriers or stressors or stimuli,” “human perception of images,” “street-level scene,” “convolutional neural networks,” “mobile transport technologies,” “older adult modes of transport.”

## Results

Our results are summarized below as three separate topics.

### Assessing Barriers and Stressors in Community Environments Through Wearable Sensing Technologies

Outdoor physical activities can be described as sleep/wake or as active/sedentary behavior (10, 11). These activities can be quantified by intensity of physical activity (12) or activity energy expenditure (13) or activities of daily living such as walking, running, sitting, stepping (14), and by using various transportation modes such bus, bicycle, car, and subway (15). The ambulatory monitoring of these measures requires comfortable, inexpensive, and accurate equipment, such as wearable devices.

The various confounding factors presented in the captured real-life data further require the contextualization of the corresponding signals, which can be achieved through location tracking with Global Positioning System coordinates (16). With recent advancements in sensing technologies, products such as Actigraph unit (4), Actical (7), Sensewear (8), and GENEActiv (17) can be drawn upon in an integrated electronic device (14). The device contains an Inertial Measurement Unit (IMU), ambient light sensor, sound detector, skin temperature sensor, and heat flux (9–11, 13). Wearable devices are generally worn on one's wrist or chest over a prolonged period of time.

Additionally, the sensors can be integrated with a smartphone-based application such as the Daynamica (15) and Discovery Tool to provide transparent data with the subjective user input *via* survey (18). For example, the Daynamica has been used to deliver personalized and context-aware interventions to app users in several research projects such as investigating the association among travel options, built and natural environments, and mood states in transport environments (15).

The physiological responses of older adult pedestrians can be reflective of human experience toward a surrounding environment, providing us unique insights into the elements of the urban built environments (e.g., neighborhood disorders and environmental barriers) (19–23). Various types of physiological response data including gait patterns, electrodermal activity (EDA), heart rate and brain activity (22, 24–26) have been have been investigated from collected physiological signals in virtual environments (27–29) naturalistic ambulatory settings and daily life locations, such as neighborhoods, downtown, urban parks, and university campuses (18, 20, 22, 23, 30–35). See Table 1 for a full list of references (18–32, 36–55). The physiological response data have been examined to recognize stress during walking trips and/or stressors of the surrounding environment on personal characteristics such as age (32, 38), gender (20), and degree of disability (37). The researchers investigated how specific populations including older adults and those visually impaired respond to the elements of urban built environment during walking trips.

**Table 1:** Summary of stressors in community environments through wearable sensing technologies and computer vision techniques for adults and for those with disability.

**Author (year)**	**Location (Country)**	**Measures**	**Stressors**
Wilhelm et al. (36); Chaspari et al. (24); Chaspari et al. (25); Saitis and Kalimeri (37); Osborne and Jones (27); Tilley et al. (38); Chrisinger and King (18); Yadav et al. (39); Can et al. (40); Hackman et al. (28); Hedblom et al. (29); Kim et al. (21); Lee et al. (41); Ojha et al. (42); Ahn et al. (22); Kim et al. (23); Lee et al. (30, 31)	United States, United Kingdom, Iceland, Switzerland	Electrodermal activity	Mild electric shocks in the virtual environments of urban parks and forests; graffiti; garbage; litter on street; lack of curb ramp; side slopes; vertical displacement; sidewalk obstructions; unpaved sidewalk; sound level; illuminance; dust in the air; relative humidity; broken house; barking dogs; uneven sidewalk; no sidewalks; tree limb; and a storage for gas container
Jebelli et al. (43); Jebelli et al. (44); Kim et al. (19); Yang et al. (45); Kim et al. (46); Yang et al. (47); Duchowny et al. (48); Kim et al. (49); Ahn et al. (26); Kim et al. (20), Kim et al. (21); Twardzik et al. (50); Yang et al. (51); Ahn et al. (22); Kim et al. (23); Bisadi et al. (52)	United States	Gait patterns	Sidewalk condition; presence of holes, sidewalk slopes, bumps, a curb cut; broken house; barking dogs; uneven sidewalk; no sidewalks; tree limb; and a storage for gas container
Wilhelm et al. (36); Goto et al. (53); Chrisinger and King (18); Can et al. (40); Hackman et al. (28); Kim et al. (20), Kim et al. (21); Ahn et al. (22); Kim et al. (23); Bisadi et al. (52)	United States, Switzerland	Electrocardiography or Photoplethysmography	Graffiti; garbage; litter on street; broken house; barking dogs; uneven sidewalks; no sidewalks; tree limb; and a storage for gas container
Li et al. (54); Jebelli et al. (55); Ahn et al. (26); Can et al. (40); Neale et al. (32)	United States, United Kingdom	Brain activity	The mood of walking paths (e.g., urban greens, urban busy, and urban quite)
Ham and Kim (56); Ham and Kim (57); Kim and Ham (58); Kim et al. (20); Kim et al. (21), Naik et al. (59)	United States	Image scores	Residential windows; graffiti; cracks on roads; vegetation; abandoned cars; garbage on the street or sidewalks; intense land uses; and traffic

In general, the gait pattern has been shown to correlate with physical barriers of urban built environments such as sidewalk defects, curbs, slopes, and holes (19–23, 43–52) (See Table 1). Signals, such as electrodermal activity (18, 21–25, 27–31, 36–42), electrocardiography or photoplethysmography (18, 20–23, 28, 36–40, 52, 53) and brain activity (26, 32, 40, 54, 55), have been separately used to understand psychological states toward stressors in relation to negative environmental stimuli (e.g., broken houses, barking dogs, and steep stairs) and the mood of walking paths such as urban busy and quiet areas (23, 30–32). Despite the premise and potential of ambulatory monitoring approaches to overcome the subjectivity related to traditional approaches (e.g., self-reporting and surveys), physiological data collected in real-life environments are confounded by various factors (e.g., weather conditions, physical movement, and the discomfort of wearing sensors) (20, 23–27). Additional testing and evaluation of such approaches is expected to provide a basis for developing a monitoring indicator of the elements of urban built environments to promote mobility for specific demographic groups (e.g., older adults, and those with disabilities).

### Assessing Barriers and Stressors in Community Environments Through Computer Vision Techniques

To understand the source of physical and emotional distress, visual data such as street-level images are effective (20, 21, 53–55). A physical appearance of urban built environments *via* street-level images can be assessed based on human perception (59). Another tool, a visual perception survey, has been utilized to assess infrastructure defects or neighborhood disorders that can negatively affect behaviors in built environments. Such a survey tool can be leveraged for assessing stressors related to older adults' mobility and the associated physical and emotional distress through computer vision techniques. Although human perception of images is subjective (60, 61), leveraging a large amount of data obtained from a web-survey in online photo-sharing ensures the robustness of using visual data to assess human perception. An example is Photo.net started in 1997 by Philip Greenspun at MIT to study the aesthetics score of images based on peer ratings (62). This peer-rating system could be used to understand stressors in community environments and to analyze their impact on human perception. In this context, scene understanding algorithms building on the computer vision techniques have been examined. An example is the prediction of the perceived safety of a street-level scene, called “Streetscore” created as a training dataset using a machine learning model (59). Another example is the random selection and ranking of several Google Street-view images from New York, Boston, Linz, and Salzburg through a pairwise comparison (63).

In case studies, large amounts of data, on 4,109 multiple generic images were extracted for semantic scene classification and ranked through 208,738 pairwise comparisons operated by 7,782 participants (64). A trained using Support Vector Regression was used to create dataset of predicted perceived safety scores based on the Google Street-view and using a human- machine scoring framework (61, 65). The performance of the predictor was evaluated by comparing it to pairwise comparison. This research showed the potential to assess a human safety perception of the street- level scene using the computer vision techniques. However, challenges remained and included the predictor potentially failing when unusual visual elements such as atypical architecture were represented in images. Pairwise comparisons from the perspective of older adults can assess urban built environments that can cause physical and emotional distress. Additionally, vector algorithms and participatory sensing-based geospatial localization can evaluate objects in urban built environments (56, 58).

In order to scale up the computational methods to map the perceived safety to the city level and/or to the global scale, convolutional neural network models have been utilized, albeit with some challenges (57, 66). For example the dataset, called “Place Pulse 2.0, containing 110,988 images with 1.17 million pairwise comparisons, and scored by 81,630 online volunteers (59) answered six perceptual dimensions: safe, depressing, boring, lively, wealthy, and beautiful. This dataset was used to train two related convolutional neural networks: (1) Street score-CNN (SS-CNN) and (2) Ranking SS- CNN (RSS-CNN). The SS-CNN was designed for binary classification to predict which image will win against another in a pairwise comparison, but this network did not consider the total ranking over all the images in the dataset. The RSS-CNN included an additional ranking sub- network resulting in the minimization of loss on pairwise classification and total ranking over the dataset but had challenges on identifying exactly what objects in scenes create the human perception.

### Assessing Barriers and Stressors in Community Environments Through Transport Technologies, Needs, and Alternative Transport Modes

The early research on utility of transport technologies, focused on understanding driving patterns of teen drivers and how to use technology-based feedback. Such feedback included, identification of risky driving behavior (e.g., hard braking, severe turns), of mobility patterns (e.g., where and when teens drove them vehicles), and of (e.g., crashes) with the aim to eventually improve driving performance and safety (67–69). The continual evolution of low-cost, small size computing platforms has created significant opportunities to develop and deploy mobile transportation technologies and has allowed for a greater understanding of mobility patterns, and transportation needs of older adults and those with disabilities. There has been at least one demonstration effort to adapt this technology to improve older driver behaviors and safety. Manser developed smartphone-based software to collect driving behavior data in real-time and to provide behavioral and safety relevant cues that targeted motoric, cognitive, and perceptual challenges experienced by older drivers (70).

The results from the demonstration project suggests smartphone technologies can be suitably adapted to address several challenges for older drivers. However, there is a need to address individual differences to a greater degree.

Additionally, there have been significant advances in using large data pools to identify, understand, and modify driver behaviors. This data is most commonly collected by sensor sets in modern vehicles, transmitted to vehicle manufacturers, and then aggregated for use by the manufacturer or a third party (e.g., Otonomo, Wejo). The full utility of this data is being explored. Early uses include facilitating municipality and state agencies' ability to identify crash hot spots and deploy engineering-based countermeasures to modify vehicle operational parameters for optimized driver/vehicle interactions, and to assess the safety impacts of infrastructure-based infrastructure-based safety countermeasures.

The transport technologies pose two specific limitations and opportunities in their ability to address the user design needs, mobility patterns and transportation needs of older drivers and for persons with disabilities. First, mobile transportation technologies, such as smartphones require calibration to vehicles, can present data quality issues due to poorly secured mounting and can often run on specific smartphone platforms. Second, these physical considerations for secured mounting can limit the extent with which the technologies can be deployed and the scope to which they can benefit drivers.

In contrast, large data pools are collecting information from millions of vehicles across the U.S. every day. This is resulting in massive quantities of data without the need to consider any physical data limitations. Although, this may seem like a suitable solution; nevertheless, large data pools explicitly omit personally identifiable information (PII) or data that may lead to PII (e.g., location tracking near client homes). Additionally, large data pools do not provide questionnaires, surveys, and focus groups for a more complete understanding of the issues associated with older drivers and/or drivers with disabilities (71, 72). When considering the limitations for each approach for the aforementioned vulnerable populations it is evident no single solution is best for addressing the critical research questions to improve driver behavior and safety. There is reason for optimism. There are some preliminary, albeit undocumented, efforts to aggregate, mine and process multiple sources of connected (e.g., smartphone, vehicle, infrastructure, and environment). Such efforts include use of multi-sensor fusion techniques (73), aggregation of spatiotemporal data using machine learning algorithms, and use of artificial intelligence for block-chain enabled intelligent internet of things (loT) architecture to reach inference by minimizing and/or eliminating the limitations of individual data sources (74–76).

In older adults and/or in those with complex conditions [e.g., those with physical/cognitive disability, loss of driving privileges require alternative transportation options to reduce caregiver burden and to maintain independence and mobility (5, 71, 72)]. Community living younger and older adults and those with disabilities need access to transportation for timely medical and preventive care (71, 72). These vulnerable populations have transport needs to stay mobile in their communities for their well-being and for improved quality-of-life. Alternative options are needed for other transport modes beyond driving. These alternates transport modes are public ground transport (bus, subway, train), paratransit (vans/shuttles), fee-for-hire transportation (e.g., Lyft, Uber, E-hail, SilverRide, GoGoGrandParent), supplemental transportation programs (STPs), Medicaid non-emergency medical transportation, social transportation, and autonomous vehicles; see Table 2 (77–90).

**Table 2:** Transport technologies, options and alternatives modes of service for older adults and for those with disability.

**Author (year)**	**Mode of transportation**	**Examples**	**Benefits / Services**	**Limitations**
Lee et al. (77); Walker et al. (78)	Next generation cars with ubiquitous or pervasive healthcare technologies	U-Cars with augmented reality	Context aware and processing capabilities, greater penetration of navigation and telematics systems, 3D visualization, wire and collision sensing technologies, driver assistance can automate driving task	More research is needed on usability, preferences, design, accessibility for older adults, will require 5G network, internet of things (loT) platform and digital city infrastructure
Taylor et al. (79); Dickerson et al. (80)	Informal Supports/Caregivers	Family members; adult grand children	Provide real-time monitoring for daily activities and routes; fully instrumented residential neighborhoods	Caregivers may have other obligations such as work, family and may not be available
Saskatoon Council on Aging (81)	Public Ground Transport	Access bus, Train, Subways, Wheelchair Taxis	Some can be free or discounted for older adults, low-cost; specific seat accommodations for wheelchair users; some buses can “kneel” closer to ground, requires older adults to be relatively mobile	Fixed routes, services may not be available all times or during holidays; distance to public station to access services may be an issue; escort may not be available
Paratransit Services (82)	Paratransit	Specially Equipped Shuttles, Vans, Microbuses, Commercial Taxi's	For older adults and those with disabilities, pick-up at doorstep, escort services available to carry items and to ensure safely return to home; available at reduced fares by public transport for area aging agencies, may be operated both publicly or privately	May be more available in urban than rural areas, low-income older adults may not be able to afford
Senior Ride Sharing (83); Vivoda et al. (84); Rosenbloom et al. (85)	Ride Sharing (Fee-for-Hire)	Uber, Lyft, E-hail, SilverRide, GoGoGrandparent	Operate *via* apps from smartphones; pick up and drop off location set by user at their time, can call driver of rideshare; older adult or disability specific services available with large companies (e.g., UberAssist); higher service for higher pay available (e.g., Lyft); inclusive, safe and low-cost with older adult specific training (e.g., SilverRide), GoGoGrandparent)	Expensive (Uber and Lyft), access in rural areas difficult due to not enough drivers; have to reserve 24 h in advance (e.g., SilverRide)
Choi et al. (86)	Supplemental Transportation Programs (STPs)	Grassroots and community based informal senior transportation services	Low-cost; highly responsive to individual needs, local transportation services,	May not be available in rural towns
Senior Ride Sharing (83)	Medicaid Non-Emergency Medical Transportation (NEMT)	Rides might be by taxi, car, van, public bus, or a subway	For those with Medicaid eligibility, will cover cost of non-emergency transport to and from medical services and appointments, do not need to have a working vehicle available in the household; good option for those unable to travel or have a physical, cognitive, mental, or developmental limitation	Will not cover non-emergency non-medical transport, which is what older adults may need for the majority of the time
Social Transportation (87)	Social Transportation	Papa	Companionship and transportation for seniors and those with disabilities; door-to-door transportation to doctor's office, drug store, grocery store, with safety and compassion as key focus.	May not be available in all cities
Harper et al. (88); Meyer et al. (89); Bergmann et al. (90)	Autonomous Vehicles (AV's)	Tesla Autopilot, Nissan ProPilot Assist, Mercedes-Benz Distronic Plus, General Motors Super Cruise	Increase in mobility for older adults with and without restrictive medical conditions, such as those with disabilities; allows for more vehicle miles traveled; higher comfort of traveling at lower prices, can increase accessibility	Challenges with moral decision making

## Discussion

Our review synthesizing emerging trends on connected technologies, such as wearable sensors, computer vision techniques, and for transport technologies to mitigate barriers and stressors to environmental stimuli to assist safe mobility for age and disability-friendly communities. Given limited reviews exist on new generation technologies, this review is timely and novel, as it synthesizes findings from multidisciplinary perspectives.

We conducted a comprehensive review on stressors and identified numerous challenges and confounders involving connected technologies and for connected data. Challenges for user studies involving older adult population include high subjectivity of self-reports, challenges with wearable technologies, and confounding factors on the signals related to their health conditions. Additional testing and evaluation of such approaches is expected to provide a basis for developing an indicator to monitor elements of the urban built environment for specific demographic groups (e.g., younger and older adults and those with disabilities) with the goal to promote older adult's mobility. Future research on assessing stressors using vision data would involve exploring involve exploring the determinants of perceptual factors of distress in community environments. In addition, there is a need for building scene-centric databases with scene categories in the context of environmental stressors causing physical and emotional distress.

We acknowledge several limitations. Despite conducting a thorough review of literature, we may have missed other relevant findings. Additional limitations may be related to location (e.g., country) and populations (e.g., age group and gender). These can minimize the generalizability of our results in different social, cultural, population, and environmental contexts.

## Conclusion

Environmental design for connected technologies and interventions to promote independence/mobility and to reduce barriers and stressors likely requires smart connected age and disability-friendly communities and cities (1, 5, 32, 71, 72, 91). Additionally, retaining older adults in a community who otherwise might leave to institutional settings can be an important economic policy and city development strategy.

## Author Contributions

PZ, JiK, JaK, MM, YH, TC, and CRA conception of mini-review, literature review, and for important intellectual content. PZ synthesized the first draft, it's revisions, and the final draft. CRA was responsible for securing funds. PZ, JiK, JaK, YH, and TC assisted with subsequent revisions. All authors contributed to the topics for this mini-review and approved the final version.

## Conflict of Interest

The authors declare that the research was conducted in the absence of any commercial or financial relationships that could be construed as a potential conflict of interest. The reviewer SZ declared a shared affiliation, though no other collaboration, with the authors to the handling Editor.
